# Effect of food processing on degradation of hexachlorocyclohexane and its isomers in milk

**DOI:** 10.14202/vetworld.2017.270-275

**Published:** 2017-03-03

**Authors:** Sujatha Singh, Krishnaiah Nelapati

**Affiliations:** Department of Veterinary Public Health and Epidemiology, College of Veterinary Science, P. V. Narsimha Rao Telangana Veterinary University, Rajendranagar, Hyderabad - 500 030, Telangana, India

**Keywords:** gas chromatography-electron capture detector, hexachlorocyclohexane isomers, pesticide residues, quick, easy, cheap, effective, rugged and safe method

## Abstract

**Aim:**

To study the effect of different food processing techniques on the degradation of organochlorine compounds (α, β, ɣ and δ hexachlorocyclohexane isomers (HCH)) residues in both natural and fortified samples of milk.

**Materials and Methods:**

Raw milk samples are collected from the local areas of Hyderabad, India. Naturally and fortified milk samples (HCH) were subjected to various food processing techniques, pasteurization (63ºC for ½ h), sterilization (121ºC for 15 min) and boiling for 5 min and analyzed by gas chromatography with electron capture detector using quick, easy, cheap, effective, rugged and safe method for multiresidue analysis of pesticides in milk with slight modification.

**Results:**

The final mean residual concentration of pesticide in milk after heat processing and percentage of degradation were calculated with respective treatments.

**Conclusion:**

Heat treatments are highly effective on reduction of mean residual concentration of HCH in milk. In which Sterilization and boiling proved to be more effective in degradation of HCH isomers.

## Introduction

Milk is highly nutritious diet for all age groups including adolescents and patients. The contamination of milk is considered as one of the major public health problems; mainly arise due to biological agents and residues of pesticides, antibiotics, and heavy metals. Injudicious and indiscriminate usage of pesticides, not only contaminate the ecosystem, but also bio-accumulate in the food chain and can be traced in plant and animal tissues causing serious health hazards [1,2]. As per World Health Organization (WHO) approximately 20,000 deaths annually were recorded, till now due to pesticide exposures in human population [3], therefore, there is strong need for discussion, on causes of environmental contamination, pesticide residues in milk, meat and other dairy products. The organochlorine (OC) compounds usage has been restricted in agriculture but permitted to use in limited quantities successively for public health activities in controlling vector-borne diseases in most of the developing countries and also in India [4].

In India, 58% of the population engaged in agriculture, so they are more exposed to the pesticides directly or indirectly. The OC insecticides known to be extremely persistent compounds are either banned or their uses are severely restricted in most of the developed countries in the world [5]. In the recent past, γ hexachlorocyclohexane (HCH) (lindane) and technical benzene hexachloride (BHC) (a mixture of HCH isomers) have been used extensively, particularly for the control of agricultural pests and mosquitoes. The use of the technical grade of HCH has been banned in India for April 1997, and only lindane can be used for field application on crops. Among various HCH isomers, only lindane has the insecticidal property and is the only isomer of HCH which is permitted for use in agriculture [6]. The range of HCH isomer concentration (as sum of each isomer) is extremely wide (0.001-4.0 mg/L) in milk and milk products in different countries [7-9]. In India, dairy milk and milk products are highly contaminated with dichlorodiphenyltrichloroethane (DDT) and HCH isomers for decades [10,11]. The OC compounds are known for inducing or aggravating certain health problems in humans such as cancer, immune systems suppression, and the disruption of hormonal functions [12]. Due to lipophilic and relative stable properties of these residues, may easily metabolized in different products and accumulate in fatty tissues of animal (meat, milk, etc.). Continuous intake of contaminated milk and milk products may lead to biomagnification of these residues in the human body, causing chronic toxicity after long-term exposure. To ensure food safety, it is necessary to find simple and cost-effective strategies to reduce pesticide residue concentration in the food commodities. Food processing is the best alternative, at domestic and industry level to tackle the current scenario of unsafe food. The adventitious removal of residues by processing is influenced by the type of food, location of pesticide, nature of pesticide, and processing method [13,14]. Food processing of livestock products, i.e., cooking, boiling, sterilization, microwave oven cooking, drying, fermentation, and storage processes exhibited large percentage of reduction in residue levels [14-16].

This study was undertaken to study the effect of different food processing methods on degradation of HCH isomers (α, β, γ and δ HCH) in both natural and fortified samples of milk.

## Materials and Methods

### Ethical approval

No animal was harmed or given stress during collection of milk samples.

### Collection of milk samples

Raw milk samples were collected from local markets of Hyderabad. Later both natural and spiked milk samples (α, β, γ and δ HCH at 1 parts per million [ppm]) were processed by adopting quick, easy, cheap, effective, rugged and safe (QuEChERS), AOAC official method 2007.01 with slight modification for analysis of pesticide residues and method was validated with recovery of 70-120% is the acceptable limit for the analysis of pesticide residues in milk samples on gas chromatography-electron capture detector (GC-ECD).

### Chemicals and reagents

Acetonitrile (ACN), acetone, n-hexane, anhydrous sodium sulfate, sodium acetate, primary secondary amines (PSA) of high-performance liquid chromatography residue grade were obtained from Qualigens and Merck Specialities Private Limited. Analytical standards with >99% purity were obtained from Dr. Ehrenstosfer, Germany and stored in deep freeze maintained at −40°C.

### Pesticide analysis

Multiresidue pesticide analysis was performed using QuEChERS method with slight modification [17,18]. Weigh 15 g of milk samples with 15 ml of ACN containing 1% acetic acid followed by addition of 6 g of anhydrous magnesium sulfate (MgSO_4_) and 1.5 g of sodium acetate. Shake vigorously for 1 min by hand. Later centrifuge the tubes at 5000 rpm for 1 min. After centrifugation, 1 ml of ACN extract will be transferred to mini centrifuge tubes for dispersive solid phase extraction, i.e. (d SPE) in which 50 mg of PSA, 50 mg of C_18_ (octadecylsilane) and 150 mg of anhydrous MgSO_4_ were added and mixed the extracts for 20 s. The tube was mixed for 30 s using a vortex mixer and centrifuged again for 5 min at 3000 rpm. Finally, 2 ml of clear extract was collected and evaporated under the gentle stream of nitrogen (15 psi) using Turbovac LV set at 52°C until near dryness up to 20 min. The dried residue content was reconstituted in 1 ml of n-hexane and further analyzed in GC-ECD set under the standard operating conditions (Table-1). In control samples, pure and fortified milk samples with standard mixtures were analyzed using the same protocol. The recovered residue levels from both natural and spiked samples of milk were calculated using following formula.

**Table-1:** GC operating conditions.

GC column	Zebron-ZB - 50, length – 30 m, 0.25 µm film thickness, internal diameter 0.25 mm
Column oven (°C)	280°C
Detector temperature	300°C
Injector temperature	260°C
Injector status	Font injector type split, split ratio 1:10
Carrier gas flow (ml/min)	12.4 ml/min
Total run time (min)	60 min

GC=Gas chromatography


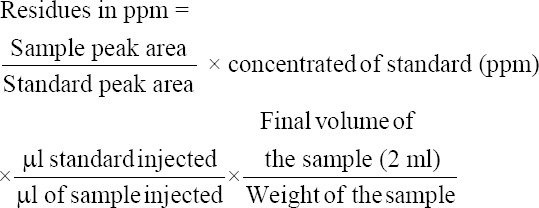


### Method validation

**Figure-1:**
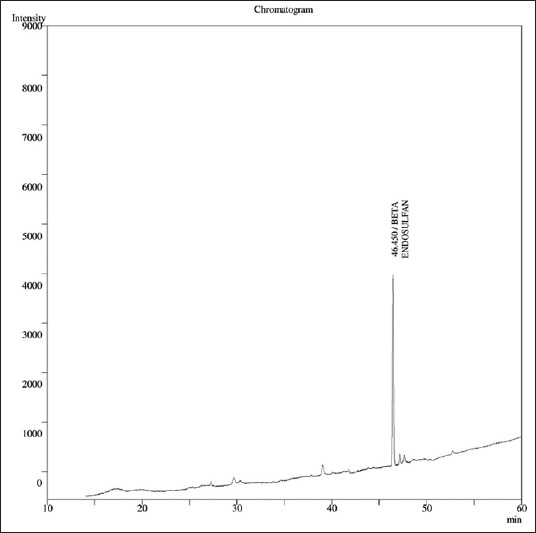
Chromatogram representing elution pattern of beta endosulfan standard.

**Figure-2:**
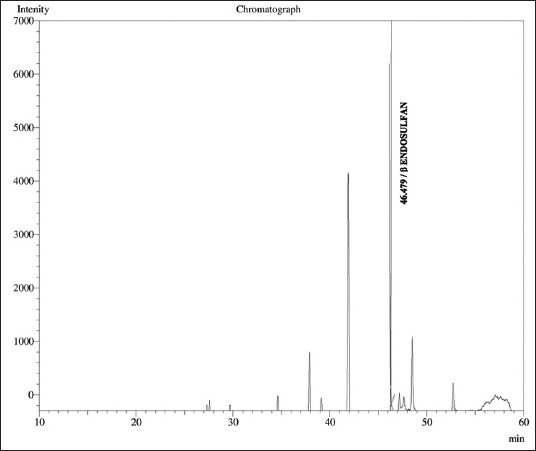
Chromatogram representing the elution pattern of beta endosulfan in fortified milk.

The required quantity of OC compounds of International Standards was prepared from certified reference materials obtained from Dr. Erhenstofer, Germany, and stock standards were obtained from All India Network Project on Pesticide Residue Lab located at Professor Jayashankar Telangana State Agricultural University, Rajendranagar, Hyderabad, Telangana, India. The beta-endosulfan standard was fortified in the representative samples of milk at the rate of 0.1 ppm. The recovery of pesticide residues above 70-120% is considered, as the acceptable limit. The pattern of elution of beta-endosulfan standards (Figure-1) was analyzed on the basis of specific retention time for GC-ECD. The limit of detection and limit of quantification of specific OC compounds were 0.01 ppm and 0.05 ppm, respectively. The recovery value was calculated from the calibration curves constructed from the concentration and peak areas of the obtained chromatograms from the milk samples (Figure-2) with standards of OC pesticide. Blank analysis of milk samples was also performed to check the different matrix interferences.

### Statistical analysis

The procedure was run thrice with each sample 10 in numbers were analyzed statistically through one-way ANOVA using SPSS software.

## Results

The mean residual levels and percentage of degradation of OC compounds after heat processing in both natural and spiked milk samples were presented in Table-2. Residual levels of α HCH content in natural raw milk was 0.0460 ppm, degraded to 0.0375, 0.0150 and 0.0275 ppm accounting 18.47%, 45.65% and 40.21% of degradation after pasteurization, boiling and sterilization, respectively. In the spiked (1 ppm) raw milk, the initial concentration was 1.11 ppm which has been degraded to 0.895, 0.3587 and 0.4377 ppm accounting 19.36%, 76.69% and 60.56% reductions, respectively. The content of α HCH in raw and spiked milk was differed significantly among the treatments. The processing methods, i.e., boiling and sterilization did not differed significantly in natural samples, whereas in spiked milk, the residual content was differed significantly (p<0.01) among the treatments. The β HCH content in natural raw milk was 0.0634 ppm, degraded during heat processing reaching 0.0532 ppm in pasteurized milk, 0.0398 ppm in boiled and 0.0235 ppm in sterilized milk, accounting 16.08%, 37.22% and 62.93%, respectively. In the spiked (1 ppm) raw milk, the initial concentration of β HCH was 0.785 ppm, degraded to 0.6712 in pasteurized, 0.4444 in boiled and 0.2745 ppm in sterilized milk accounting to 14.49%, 43.38% and 65.03% reductions, respectively. The content of β HCH in raw and spiked milk was differed significantly (p<0.01) among the treatments in both natural and spiked milk samples.

**Table-2:** Residual levels of organochlorine compounds after heat processing in milk.

Name of the pesticide	Raw milk (ppm)	Pasteurization (ppm)	Deg (%)	Boiling (ppm)	Deg (%)	Sterilization (ppm)	Deg (%)
α HCH							
Natural	0.0460±0.005^a^	0.0375±0.003^b^	18.47	0.0150±0.0025^c^	45.65	0.0275±0.002^c^	40.21
Spiked	1.11±0.023^a^	0.895±0.019^b^	19.36	0.3587±0.002^d^	76.69	0.4377±0.013^c^	60.56
β HCH							
Natural	0.0634±0.003^a^	0.0532±0.025^b^	16.08	0.0398±0.021^c^	37.22	0.0235±0.012^d^	62.93
Spiked	0.785±0.05^a^	0.6712±0.039^b^	14.49	0.4444±0.006^c^	43.38	0.2745±0.010^d^	65.03
γ HCH							
Natural	0.0158±0.002^a^	0.0140±0.002^b^	11.39	0.0115±0.03^c^	27.21	BDL	-
Spiked	0.756±0.05^a^	0.5676±0.024^b^	24.92	0.3605±0.016^c^	52.31	0.2883±0.019^d^	61.86
δ HCH							
Natural	0.4732±0.04^a^	0.4327±0.014^b^	8.5	0.312±0.015^c^	34.06	0.256±0.012^d^	45.90
Spiked	0.854±0.06^a^	0.745±0.058^b^	11.70	0.355±0.014^c^	58.43	0.254±0.014^d^	70.25
Total HCH							
Natural	0.5984±0.032^a^	0.5374±0.017^b^	10.19	0.3737±0.016^c^	37.55	0.307±0.018^d^	48.69
Spiked	3.505±0.65^a^	2.878±0.51^b^	17.88	1.618±0.22^c^	66.85	1.221±0.21^c^	65.16

Residual levels bearing different superscripts (a, b, c, and d) horizontally differed significantly (p<0.01). HCH=Hexachlorocyclohexane

The γ HCH content in natural raw milk was 0.0158 ppm, degraded during heat processing reaching 0.0140 ppm in pasteurized milk and 0.0115 ppm in boiled, accounting 11.39 and 27.21% of degradation, respectively, whereas it was below detectable level after sterilization. In the spiked (1 ppm) raw milk, it was 0.756 ppm, degraded to 0.5676 in pasteurized milk, 0.3605 ppm in boiled milk and 0.2883 ppm in sterilized milk accounting 24.92%, 52.31% and 61.86% reductions, respectively. The content of γ HCH in raw and spiked milk differed significantly with the treatments, and all the treatments differed significantly (p<0.01) among themselves.

The δ HCH content in natural raw milk was 0.4732 ppm, reduced after heat processing reaching 0.4327 ppm in pasteurized milk, 0.312 ppm in boiled, 0.256 ppm in sterilized milk accounting 8.5%, 34.06% and 45.90% reductions, respectively. In the spiked (1 ppm) raw milk, it was 0.854 ppm, degraded to 0.745 ppm in pasteurized milk, 0.355 ppm in boiled milk and 0.254 ppm in sterilized milk accounting 11.70%, 58.43% and 70.25% reductions, respectively. In natural and spiked milk samples, the residue levels differed significantly (p<0.01) and also among the three treatments.

The total HCH content in natural raw milk was 0.5984 ppm, reduced to 0.5374 ppm in pasteurized milk, 0.3737 ppm in boiled, 0.307 ppm in sterilized milk accounting 10.19%, 37.55% and 48.69%, respectively. In the spiked (1 ppm) raw milk, the total HCH was 3.505 ppm, degraded to 2.878 ppm in pasteurized milk, 1.618 ppm in boiled milk and 1.221 ppm in sterilized milk accounting 17.88%, 66.85% and 65.16% reductions, respectively. In natural and spiked milk samples, the residue levels differed significantly (p<0.01) within treatments, whereas in spiked milk boiling and sterilization treatments did not differ significantly.

## Discussion

During food processing the pesticide on the basis of individual nature, may degrade in different ways, such as it may evaporate, thermally degrade or co-distillate accordingly [16]. Food processing studies are very important, to relate the levels of residue concentration in raw food commodities to the level of processed food products, to evaluate the strength of processing factor in degradation of various pesticide in the products [19]. Most of the OC compounds and its metabolites are lipid soluble, and thus they found more concentrated in the fatty portions of foods especially in butter, cream ghee and cheeses, etc. [10,20]. The percentage of degradation in both natural and spiked samples of milk was (18.47% and 19.36% for α HCH), (16.08% and 14.49% for β HCH), (11.39% and 24.92% for γ HCH), (8.5% and 11.70% for δ HCH), and (total HCH 10.19% and 17.88%), respectively, on pasteurization. Pasteurization could not eliminate the residues in the milk, but degraded to some extent which was similar to 15.38% of degradation [7] whereas very little effect of pasteurization seen on the degradation levels of hexachlorobenzene (HCB) and lindane content in milk samples [21] whereas some scientist reported that HCB pesticide residue remained unaffected by pasteurization [22]. The percentages of degradation in both natural and spiked samples of milk were 45.65% and 76.69%, 37.22% and 43.38%, 27.21% and 52.31%, 34.06% and 58.43%, 37.55% and 66.85%, respectively, for α, β, γ and δ HCH. During boiling, the milk samples were subjected to higher temperature where considerable decrease in residue levels in all isomers of HCH were recorded, while in β HCH degradation was less compared to other isomers of HCH in spiked samples, as by the physiochemical nature of β HCH have lower volatility and high melting point, makes more stable compound than rest of the isomers of HCH [23]. The percentage of degradation of 24.49% was seen in β HCH content on boiling, which was less than the present study [24]. Skim milk recombined with butter oil fortified with 2 ppm HCH shown very little impact of boiling and boiling with malai removal reduced the HCH isomers by 11.54-26.78% and 35.86-50.88% [25]. Boiling for 5, 10, and 15 min reduced the lindane levels by 75.0%, 79.6% and 85.4%, respectively [26]. Spiked samples of milk with lindane subjected to boiling for 2 min and reported that boiling was more effective in dissipation of pesticide residues [27]. The percentage of degradations in both natural and spiked samples of milk on sterilization were 40.21 and 60.56 for α HCH, 62.93 and 65.03 for β HCH, 61.86 for γ HCH, 45.90 and 70.25 for δ HCH and 48.69 and 65.16 for total HCH, respectively. Sterilization of milk at 12ºC for 15 min. showed 83.25%, 91.67% and 68.70% loss in β BHC, lindane and p, p’ DDT content, respectively [28]. Skim milk recombined with butter oil fortified with 2 ppm HCH on sterilization, there was 19% of degradation in the HCH content [29]. The effect of sterilization on the residues of lindane and its metabolites contaminated at the level of 1 mg/kg fat, observed to reduce the residual content by 84.4% and 76.6%, respectively [26].

## Conclusion

The HCH residues were detected in the milk samples, which exceeded the maximum residue level limits (0.001 ppm), recommended by FAO/WHO. Effect of different food processing plays an efficient role on degradation of OC compound residues in milk. Most of the residues, which are highly lipophilic nature, are hardly get metabolized, hence it may easily concentrate in milk products leading to bioaccumulation and biomagnification [30]. Prolonged consumption of contaminated milk will severely affect the human health and responsible for producing chronic effects like cancer and risk of Alzheimer’s disease [31]. Application pesticide in enhancing agricultural productivity must be balanced against possible health hazards arising from toxic pesticide residues in food. First and foremost thing is application pesticide should be in compliance with Good Agricultural Practices and shifting from chemical farming to organic farming. However, in developing countries like India, the process of adoption of good agricultural practices and organic farming is very slow; meanwhile, it is highly practical to adopt some simple processing techniques and acquire significance for reducing the harmful pesticide residues in food.

## Authors’ Contributions

The study was part of SS’s research work during the Ph.D. program. SS prepared, processed and analyzed the samples. KN designed and planned the research programme and approved the final manuscript.
